# Interleukin 32 Expression in Mesothelioma

**DOI:** 10.1111/1759-7714.70353

**Published:** 2026-07-15

**Authors:** Takuya Mikamo, Riko Niwa, Yuki Hanamatsu, Tamotsu Takeuchi

**Affiliations:** ^1^ Department of Pathology and Translational Research Gifu University Graduate School of Medicine Gifu Japan; ^2^ Department of Pathology Matsunami General Hospital Gifu Japan; ^3^ Center for Translational Research Matsunami General Hospital Gifu Japan

**Keywords:** carcinogenesis, IL32, immunohistochemistry, mesothelioma

## Abstract

**Background:**

Interleukin 32 (IL32) has context‐dependent roles in carcinogenesis across cancer types. In this study, we examined IL32 in mesotheliomas.

**Methods:**

IL32 protein expression was evaluated by immunohistochemistry in 56 mesothelioma tissue microarray specimens and by immunoblot analysis in cultured mesothelioma cell lines. The functional roles of IL32 were examined through ectopic expression of IL32β and IL32θ isoforms in D‐Meso‐Sonobe cells with epithelial‐mesenchymal plasticity and through siRNA‐mediated IL32 downregulation followed by cell‐detachment‐induced apoptosis assays in epithelioid mesothelioma cells.

**Results:**

IL32 immunoreactivity was detected in 20 of 56 mesothelioma tissue specimens, including cytoplasmic staining in 20 of 39 epithelioid cases, whereas little or no immunoreactivity was found in 17 sarcomatoid cases. In immunoblot analysis, an IL32 protein band was detected in two cultured epithelioid mesothelioma cell lines (MPM‐2 and TCC‐Meso‐1), but not in sarcomatoid MPM‐1 cells. Two IL32 isoforms, IL32β and IL32θ cDNAs, were isolated from MPM‐2 cells. Ectopic expression of IL32θ, but not IL32β, inhibited the morphological transition from epithelioid to sarcomatoid features in D‐Meso‐Sonobe mesothelioma cells with mesothelial‐mesenchymal transition plasticity. Conversely, siRNA‐mediated downregulation of IL32 increased cell detachment‐induced apoptosis in MPM‐2 and TCC‐Meso‐1 epithelioid mesothelioma cells. Moreover, IL32 suppressed expression of secreted protein acidic and rich in cysteine, which is an epithelial‐mesenchymal transition‐related protein in mesothelioma cells.

**Conclusions:**

These findings indicate that IL32 is expressed in epithelioid mesothelioma and may contribute to mesotheliomagenesis.

## Graphical abstract

IL32θ suppresses epithelial‐mesenchymal transition in mesothelioma cells. In D‐Meso‐Sonobe cells, which exhibit epithelial‐mesenchymal plasticity, forced expression of IL32θ attenuates the spindle‐shaped morphology (left) and promotes a more epithelioid phenotype (right).

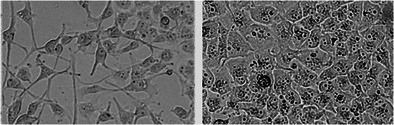

## 1 Introduction

Invasive diffuse mesotheliomas are histopathologically classified into three major subtypes: epithelioid, sarcomatoid, and biphasic mesothelioma [1]. Historically, sarcomatoid mesothelioma has been considered to be associated with a less favorable prognosis than epithelioid mesothelioma [2], which has been attributed to high proliferative activity and weak responsiveness to chemotherapy in sarcomatoid mesothelioma cells. The prognosis of biphasic mesothelioma may depend on the ratio of epithelioid to sarcomatoid cells, with a higher proportion of sarcomatoid cells linked to poorer survival [3]. Clarifying the molecular mechanisms involved in epithelial‐mesenchymal transition (EMT), or mesothelial‐mesenchymal transition, may help develop novel therapies to improve patient outcomes.

Epithelioid mesothelioma is the most common type, accounting for approximately 70% of diffuse mesothelioma cases. Although it is the least aggressive histopathological subtype, the median overall survival is less than 2 years. Surprisingly, the apoptotic index of epithelioid mesothelioma is lower than that of sarcomatoid mesothelioma [4]. Cancer cells are generally susceptible to cell‐detachment‐induced apoptosis (anoikis); however, epithelioid mesothelioma cells are often highly resistant to anoikis in pleural effusion or ascites [5]. Most patients with pleural epithelioid mesothelioma require management of malignant pleural effusion (MPE). In vitro studies have suggested that MPE may promote tumor cell growth in mesothelioma [6]. Collectively, these findings support the need to understand the molecular mechanisms underlying resistance to cell‐detachment‐induced apoptosis in epithelioid mesothelioma cells.

Interleukin 32 (IL32) is a non‐canonical cytokine [7]. For example, IL32 lacks structural similarity to other cytokines and, therefore, does not belong to the cytokine superfamily. In general, interleukins have few, if any, alternatively spliced isoforms. Conversely, IL32 has nine splice isoforms, each of which may exhibit distinct biological properties. IL32γ, which is one of the nine IL32 isoforms, can be secreted because the IL32γ–specific exon encodes a potential signal peptide. By contrast, other IL32 isoforms do not contain a canonical hydrophobic signal peptide at the N‐terminus. Additionally, a specific IL32 receptor that mediates extracellular signaling has not yet been identified.

Several studies have reported that IL32 acts as an intracellular mediator affecting pleiotropic molecular pathways involved in cell growth and death. IL32 is expressed in the cytoplasm of several malignant tumors [8, 9]. It enhances TNF‐α‐induced colon cancer cell death in vitro and tumor regression in vivo [10]. Additionally, IL32 inhibits lung cancer stem cell proliferation [11]. Conversely, it promotes breast cancer cell growth and invasiveness [12] and increases human gastric cancer cell invasion [13]. IL32 functions as a tumor suppressor or activator may vary depending on the expressed isoform and cancer cell type [14, 15].

The pathobiological properties of IL32 in mesotheliomagenesis remain unclear. A previous study found IL32 transcripts in cultured mesothelioma cell lines [16]. Nonetheless, IL32 protein expression in mesothelioma cells has not yet been fully characterized. Together with recent findings that proteasome degradation regulates IL32 in malignant cells [17, 18], clarification of IL32 protein expression in mesothelioma is warranted. In this study, we aimed to determine IL32 protein expression in mesothelioma tissues and subsequently to explore its pathobiological roles using cultured mesothelioma cells.

## 2 Methods

### 2.1 Tissue Specimens and Immunohistochemical Staining

Tissue microarrays of mesothelioma (Cat. No. MS1001b) were purchased from US Biomax (Rockville, MD, USA). Tissue specimens were collected under the Health Insurance Portability and Accountability Act (USA) with informed consent from the donors and in accordance with ethical standards and the Health Insurance Portability and Accountability Act. Commercially available tissue microarrays were used for immunohistochemical staining, thereby avoiding patient‐selection bias.

Immunohistochemical staining was performed as previously reported [19, 20]. Briefly, deparaffinized sections were autoclaved for 15 min in 10 mM citrate buffer (pH 6.0). The slides were then incubated for 30 min with normal horse serum. Next, the tissues were incubated with a 1:200‐diluted goat anti‐IL32 antibody (Cat. No. AF3040; R&D Systems, Minneapolis, MN, USA), followed by incubation with ImmPRESS Excel‐amplified HRP polymer reagent (anti‐goat IgG) (Vector Laboratories Inc., Burlingame, CA, USA). Staining was considered positive when > 10% of cells exhibited immunoreactivity.

### 2.2 Cells and Culture

The MPM‐1 sarcomatoid mesothelioma and MPM‐2 epithelioid mesothelioma cell lines were established and maintained in our laboratory [21]. The biphasic mesothelioma cell line, MPM‐3, was also established in our laboratory [21]. Other epithelioid mesothelioma cell lines, TCC‐Meso‐1 [22], and the biphasic mesothelioma cell line, TCC‐Meso‐3 [22], were obtained from the Japan Health Science Research Resources Bank (Osaka, Japan).

ACC‐Meso‐1 cells were obtained from the RIKEN BioResource Research Center (Ibaraki, Japan). The ACC‐Meso‐1 cell line was originally derived from a patient with pleural epithelioid mesothelioma [23]. Nonetheless, ACC‐Meso‐1 has also been reported to exhibit a mesenchymal phenotype, with low E‐cadherin expression and high vimentin and ZEB1 expression [24].

D‐Meso‐Sonobe, which exhibits epithelial‐mesenchymal plasticity (EMP), that is, transition activity between epithelioid and sarcomatoid features, was also established in our laboratory [25, 26].

Cells were cultured in Dulbecco's modified Eagle's medium (DMEM) (Gibco Life Technologies, Grand Island, NY, USA) supplemented with 10% heat‐inactivated fetal bovine serum and without antibiotics. The cells were passaged for no longer than 6 months after resuscitation.

### 2.3 Immunoblotting

Immunoblotting was performed according to the method described by Towbin et al. with modifications as previously described [27, 28]. Briefly, cell lysates were separated by sodium dodecyl sulfate‐polyacrylamide gel electrophoresis and electroblotted onto polyvinylidene difluoride membranes (Immobilon‐P Transfer Membrane; Millipore, Bedford, MA, USA). Membranes were blocked with Block Ace (blocking milk; Yukijirushi, Sapporo, Japan) and incubated with a goat anti‐IL32 antibody (R&D Systems). For immunodetection, a donkey peroxidase‐conjugated anti‐goat secondary antibody (1:5000; cat. no. V8051; Promega, Madison, WI, USA) was used. Secreted protein acidic and rich in cysteine (SPARC) protein expression was also examined using a mouse monoclonal antibody (Cat. no. 66426‐1‐IG; Proteintech Group Inc., Rosemont, IL, USA) and a HRPO‐linked anti‐mouse IgG secondary antibody (1:5000; Cat. no. 7076; Cell Signaling Technology, Danvers, MA, USA). The membrane was developed using ImmunoStar LD Chemiluminescence Reagent (FUJIFILM Wako Pure Chemical Corporation, Tokyo, Japan), and signals were detected with an Invitrogen iBright 1500 gel imaging system (Thermo Fisher Scientific, Waltham, MA, USA). Input proteins were evaluated using a rabbit monoclonal anti‐GAPDH antibody (cat. no. 5174; Cell Signaling Technology, Danvers, MA, USA) or a rabbit anti‐intelectin‐1/2 antibody (Cat. no. 11770‐1‐AP; Proteintech Group Inc., Rosemont, IL, USA).

### 2.4 Small Interfering (si)RNA‐Mediated Gene Silencing

The detailed procedure for siRNA silencing of a target gene has been described previously [29]. siRNAs were used to silence the IL32 gene (cat. no. AM16708; siRNA IDs 140003 and 259739; Thermo Fisher Scientific Inc., Cleveland, OH, USA), whereas a Trilencer‐27 Universal scrambled negative control siRNA duplex (OriGene, Rockville, MD, USA) was used as the non‐silencing control. siRNAs were transfected into cells using Lipofectamine RNAiMAX (Invitrogen, Carlsbad, CA, USA) according to the manufacturer's instructions. Cell images were obtained using a CytoWatcher (ATTO, Tokyo, Japan) 72 h after transfection.

### 2.5 Reverse Transcriptase‐Polymerase Chain Reaction, Plasmids, and Transfection

Reverse transcriptase‐polymerase chain reaction (RT‐PCR) was performed as previously described [30]. Briefly, total cellular RNA was extracted from MPM‐2 cell lysates using RNA‐zol B (Biotex Laboratory, Houston, TX, USA). cDNA was synthesized from total RNA, and subsequent PCR was performed using the PrimeScript RT‐PCR Kit (Takara, Ohtsu, Japan). Human full‐length IL32 cDNA was amplified using the following primers: sense, 5′‐GCCATGTGCTTCCCGAAGGTCCTCTCTG‐3′; antisense, 5′‐AGGGCAAAGGTGGTGTCAGTATCTTCATTTTG‐3′. The cDNA was cloned into the pTargeT mammalian expression vector (Promega, Madison, WI, USA), transformed into JM109 cells (Takara), confirmed by sequencing, and transfected into cells.

Two weeks of selection in the presence of 500 μg/mL of G418 (Gibco BRL) were followed by isolation of individual clones. Cell clones transfected with the empty vector alone were established in parallel.

### 2.6 Quantitative RT‐PCR

Quantitative RT‐PCR was performed using the SYBR Green reaction kit following the manufacturer's protocol (Roche Diagnostics, Mannheim, Germany) in a LightCycler (Roche Diagnostics), as previously reported [29]. The following primers were used: secreted protein acidic and rich in cysteine (SPARC)‐forward 5′‐GCTGGATGAGAACAACAC‐3′; SPARC‐reverse 5′‐AAGAAGTGGCAGGAAGAG‐3′; GAPDH‐forward 5′‐GAAATCCCATCACCATCTTCCAGG‐3′; GAPDH‐reverse 5′‐GAGCCCCAGCCTTCTCCATG‐3′. The expression of each target gene was analyzed using the 2^−ΔΔCT^ method [31] embedded in the LightCycler system. ΔCT values for each gene of interest were normalized to the GAPDH values for each triplicate. Standard deviations were then calculated for each triplicate, and the fold change for each of the three target genes was recorded. The value for each of the groups (*n* = 3) was calculated as the fold change relative to the mean value for the control siRNA‐treated group (control set to 1.0).

### 2.7 Cell‐Detachment‐Induced Apoptosis Assay and Detection of Apoptosis

Cells were detached from tissue‐culture flasks using 0.25% trypsin. After detachment, the cells were seeded in a six‐well plate (1 × 10^6^/well) with an ultra‐low‐attachment surface to prevent adherence and were cultured in DMEM containing 10% FBS (Corning Life Sciences, Teterboro, NJ, USA). After 72 h of suspension culture, the cells were collected.

Apoptotic cells were quantified using fluorescein isothiocyanate‐conjugated Annexin V and propidium iodide (PromoCell GmbH, Heidelberg, Germany), as previously reported [30].

## 3 Results

### 3.1 Immunoreactivity for IL32 Was Observed in Epithelioid Mesothelioma, but Not in Sarcomatoid Mesothelioma

**FIGURE 1:**
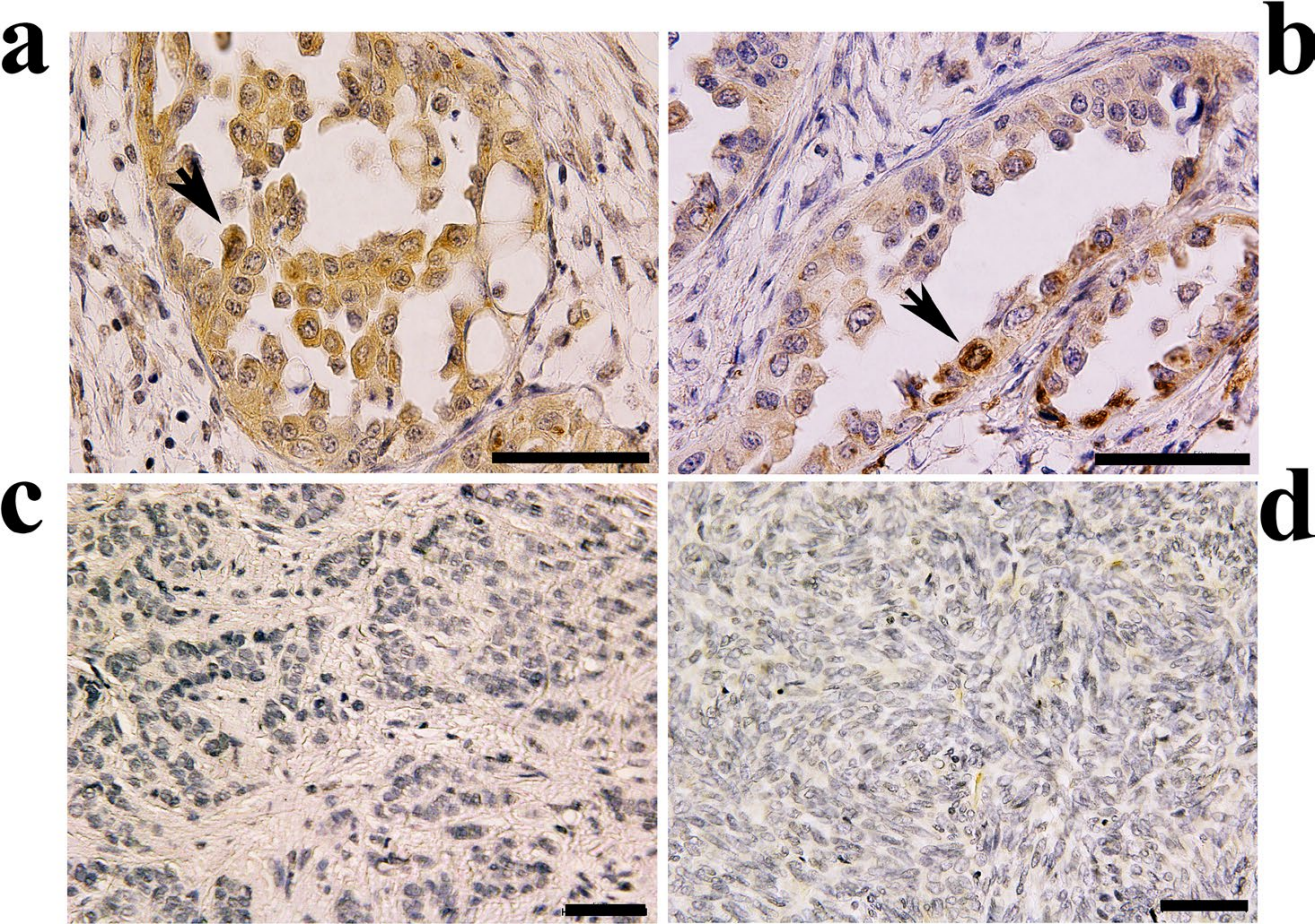
Representative immunohistochemical staining results. IL32 immunoreactivity was observed in 20 of 56 mesothelioma tissue specimens. IL32 immunoreactivity‐positive mesothelioma was an epithelioid mesothelioma with a papillotubular growth pattern (a, b). IL32 immunoreactivity was predominantly cytoplasmic, with nuclear staining also detected in several mesothelioma cells (arrows). Little IL32 immunoreactivity was identified in solid or trabecular epithelioid mesotheliomas (c). None of the 17 sarcomatoid mesothelioma tissue specimens exhibited IL32 immunoreactivity (d). Scale bar, 50 μm.

Representative immunohistochemical staining results using a specific anti‐IL32 antibody are displayed in Figure 1. IL32 immunoreactivity was observed in 20 of 39 epithelioid mesothelioma cases. Notably, papillotubular mesotheliomas exhibited strong IL32 immunoreactivity (Figure 1a,b), unlike solid or trabecular mesotheliomas (Figure 1c). Conversely, none of the 17 sarcomatoid mesothelioma tissue specimens exhibited IL32 immunoreactivity (Figure 1d).

### 3.2 IL32 Was Expressed in Cultured Epithelioid Mesothelioma Cells, but Not in Sarcomatoid Mesothelioma Cells

**FIGURE 2:**
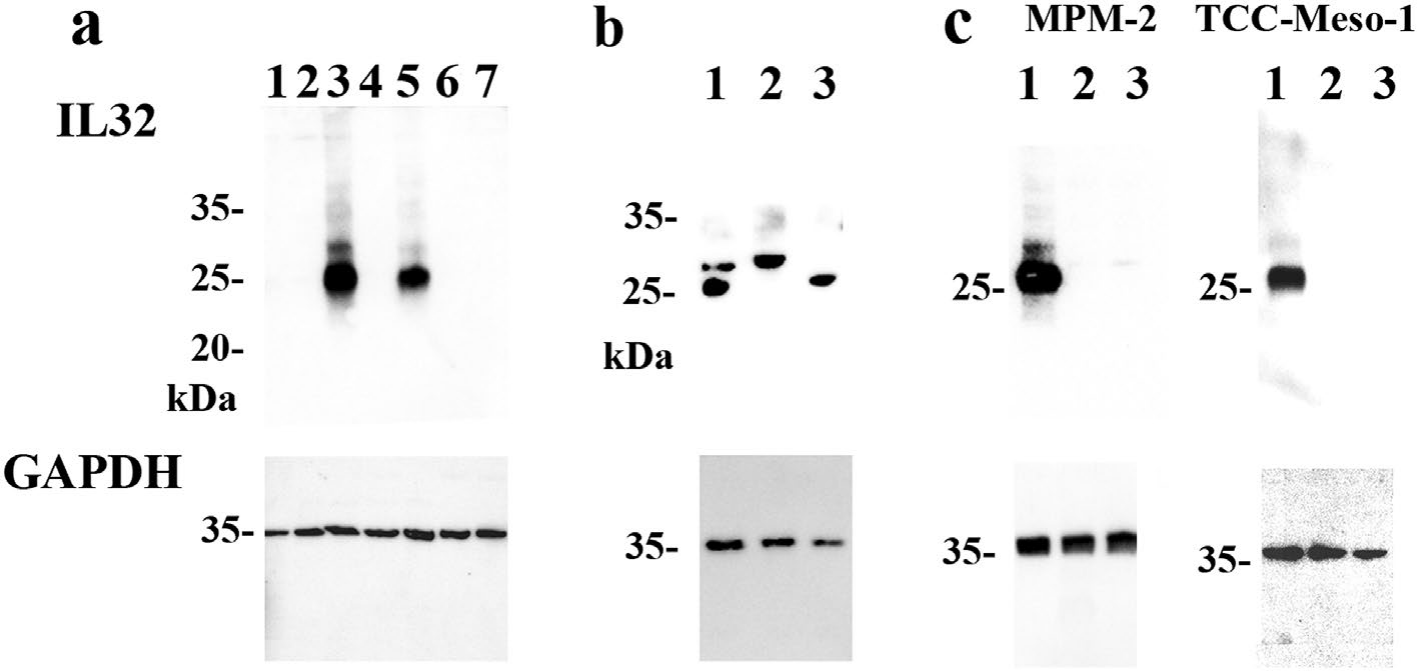
IL32 immunoblot analysis of cultured mesothelioma cells. (a) Cell lysates from ACC‐Meso‐1 (lane 1), MPM‐1 (lane 2), MPM‐2 (lane 3), MPM‐3 (lane 4), TCC‐Meso‐1 (lane 5), TCC‐Meso‐3 (lane 6), and D‐Meso‐Sonobe (lane 7) cells were subjected to immunoblotting using a specific antibody to IL32. Robust IL32 protein bands were detected in MPM‐2 and TCC‐Meso‐1 epithelioid mesothelioma cell lysates. Protein loading of cell lysates was monitored using anti‐GAPDH antibody. (b) Immunoblot analysis demonstrated two IL32 isoform bands in MPM‐2 (lane 1). Ectopically expressed IL32β (lane 2) and IL32θ (lane 3) protein bands were detected in an expression‐vector‐transfected D‐Meso‐Sonobe cell clone. (c) siRNA‐mediated downregulation of IL32 markedly diminished IL32 bands in MPM‐2 and TCC‐Meso‐1 cells (lane 1, Mock; lane 2, ID 140003; lane 3, 259739 siRNAs).

Next, IL32 expression was examined in cultured mesothelioma cells. Immunoblot analysis revealed an IL32 protein band in cultured MPM‐2 and TCC‐Meso‐1 epithelioid mesothelioma cells, but not in sarcomatoid mesothelioma MPM‐1 cells. Subsequently, IL32 cDNAs were obtained from MPM‐2 epithelioid mesothelioma cells, ligated into an expression vector, and transfected into JM109 cells. Sequencing analysis revealed that MPM‐2 epithelioid mesothelioma expressed the IL32β and IL32θ transcripts, whereas no other IL32 alternative splicing forms were identified among more than 20 transfected JM109 colonies. Immunoblot analysis also demonstrated IL32 bands at molecular weights corresponding to IL32β and IL32θ proteins in both MPM‐2 and TCC‐Meso‐1 cells (Figure 2). No IL32 protein band was detected in D‐meso‐Sonobe mesothelioma cells, which exhibit EMP. Additionally, no IL32 protein band was detected in ACC‐Meso‐1 cells, which exhibit the mesenchymal phenotype (fibroblast‐like morphology with low E‐cadherin/high vimentin and ZEB1 expression) [24]. Representative immunoblot data are presented in Figure 2 and Figure S1.

### 3.3 Enforced IL32 Expression Impaired EMP of the D‐Meso‐Sonobe Mesothelioma Cells

D‐Meso‐Sonobe is a cultured mesothelioma cell line with EMP [25, 26]. D‐Meso‐Sonobe cells transfected with an empty vector exhibited epithelioid features during passage culture at approximately 60% confluence but became spindle‐cell mesenchymal cells during subsequent continuous culturing, as reported for the original D‐Meso‐Sonobe cells [26].

**FIGURE 3:**
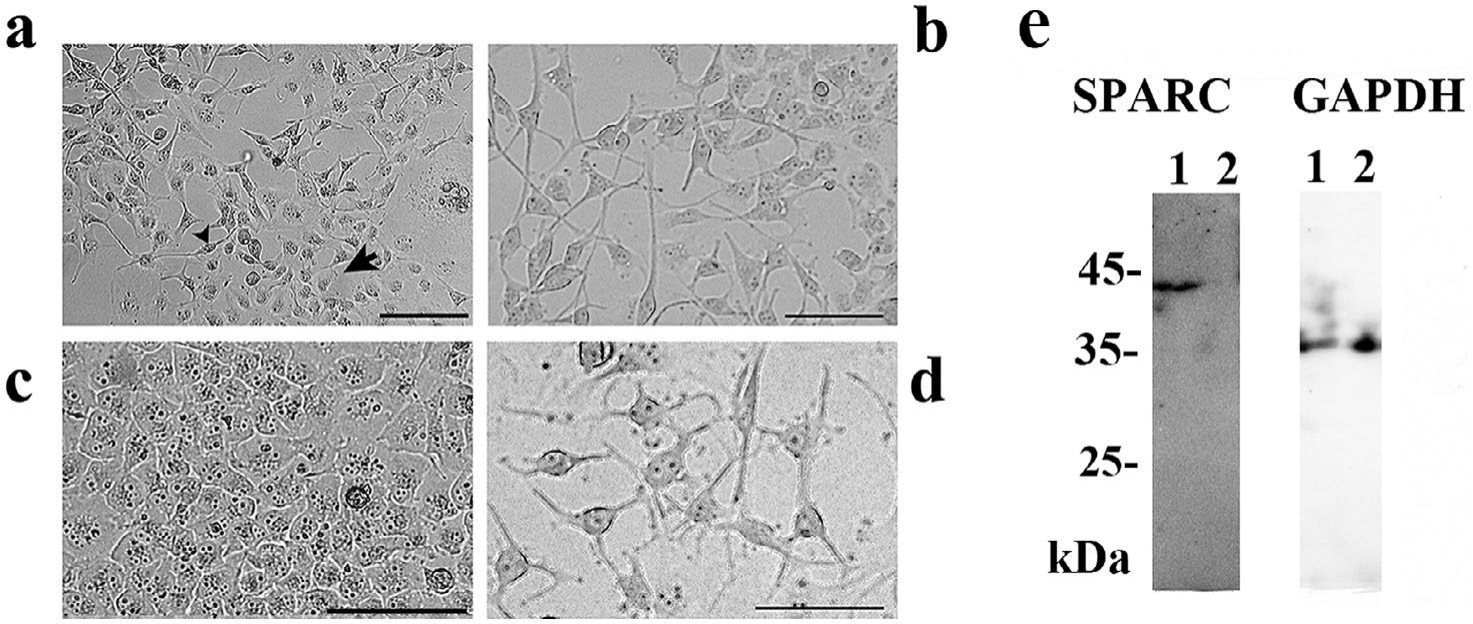
Forced IL32θ, but not IL32β, expression inhibited epithelial‐mesenchymal transition of mesothelioma cells. D‐Meso‐Sonobe mesothelioma cells exhibit epithelial–mesenchymal plasticity resembling EMT in mesothelioma. Once cultures reached > 60% confluence, empty‐vector‐transfected cells shifted from an epithelioid morphology (arrow) to mesenchymal spindle‐shaped cells (arrowhead) (a). With continued culture without passage, most empty‐vector‐transfected cells adopted a mesenchymal spindle‐cell morphology (b). In contrast, IL32θ‐expressing D‐Meso‐Sonobe cells retained an epithelioid polygonal morphology even after reaching confluence (c). IL32β‐expressing D‐Meso‐Sonobe cells underwent epithelial–mesenchymal transition similar to that observed in empty‐vector‐transfected cells (d). Scale bar, 50 μm (a), 10 μm (b–d). A SPARC protein band was detected in empty‐vector‐transfected D‐Meso‐Sonobe cells (e: Lane 1), whereas little or no SPARC band was detected in IL32θ‐expressing D‐Meso‐Sonobe cells (e: Lane 2). GAPDH served as the loading control.

Following transfection of D‐Meso‐Sonobe mesothelioma cells with an IL32β or IL32θ expression vector, several distinct clones expressing IL32 protein were obtained (Figure 2). Notably, all IL32θ‐expressing D‐Meso‐Sonobe cells retained epithelioid features even after continuous culture at full confluence. Conversely, empty‐vector‐transfected cells and IL32β vector‐transfected cells exhibited sarcomatoid features after continuous culture at full confluence, similar to the original D‐Meso‐Sonobe cells [26]. SPARC protein was expressed in empty‐vector‐transfected cells but not in IL32θ‐expressing D‐Meso‐Sonobe cells. Representative results are shown in Figure 3.

### 3.4 Downregulation of IL32 Expression Promoted Cell‐Detachment‐Induced Apoptosis in Mesothelioma Cells

**FIGURE 4:**
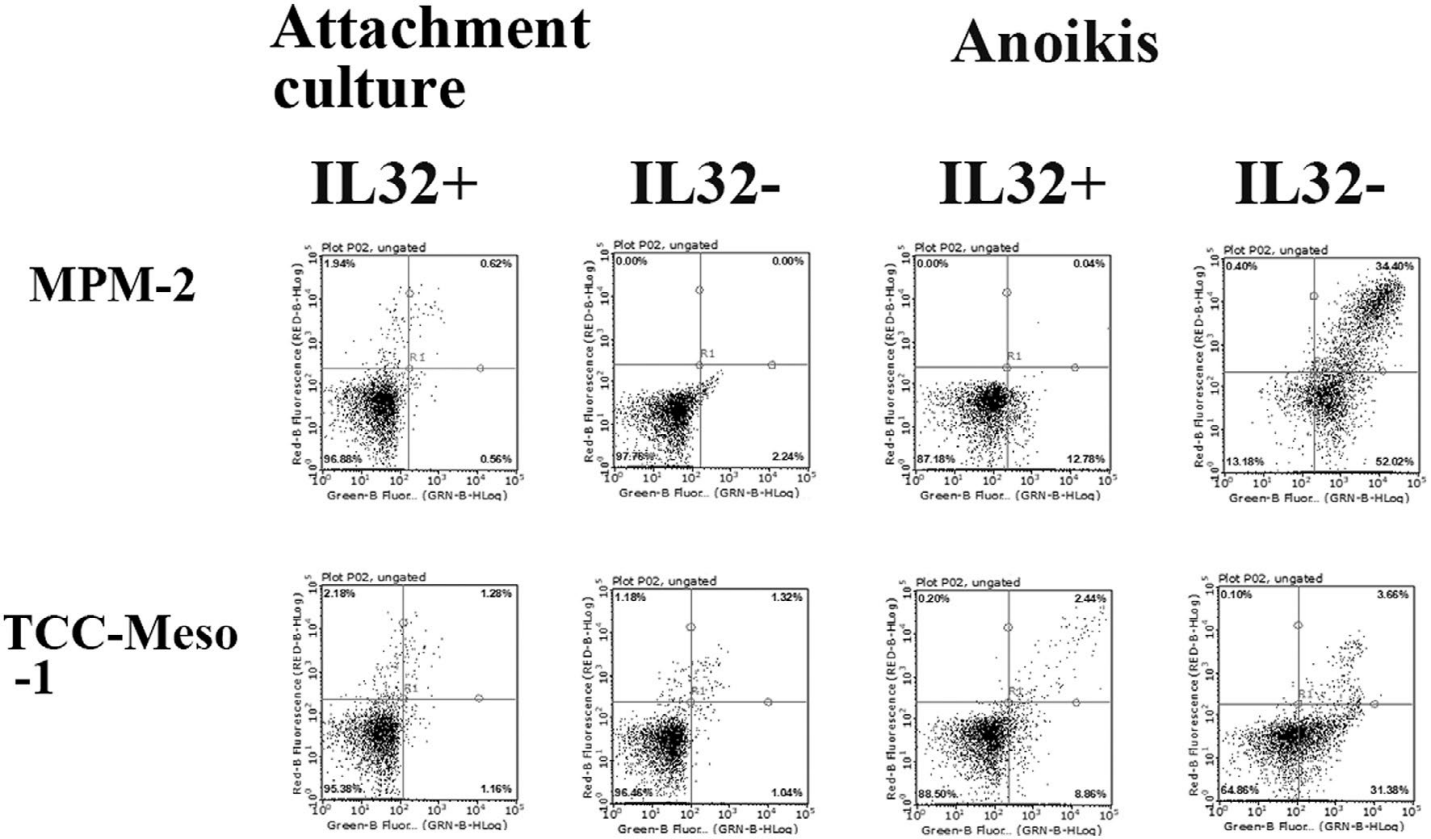
IL32 downregulation decreased epithelioid mesothelioma cell resistance to anoikis (detachment‐induced cell death). Neither control siRNA‐treated IL32‐expressing nor siRNA‐mediated IL32 downregulated MPM‐2 or TCC‐Meso‐1 cells did not induce apoptosis under attachment culture conditions. In detached cultures, IL32 downregulation (IL32‐) sensitized cells to apoptosis. Anoikis assay results for control siRNA‐treated cells are indicated as IL32+. Representative results obtained using ID 140003 siRNA are shown; similar results were obtained using another siRNA targeting IL32 (259739 siRNAs).

We next assessed whether IL32 expression influences the susceptibility of cultured epithelioid mesothelioma cells to anoikis, a form of anchorage‐dependent apoptosis, using Annexin V/PI staining. Under adherent conditions, siRNA‐mediated IL32 downregulation did not induce apoptosis in MPM‐2 or TCC‐Meso‐1 cells. In contrast, under detached conditions, IL32 downregulation increased apoptosis in both cell lines. These findings suggest that IL32 promotes anoikis resistance in epithelioid mesothelioma cells. Representative data are shown in Figure 4.

### 3.5 Downregulation of IL32 Expression Increased SPARC Expression at the Transcriptional Level

Quantitative RT‐PCR analysis demonstrated that IL32 downregulation increased *SPARC* transcript expression in MPM‐2 epithelioid mesothelioma cells. Subsequent immunoblotting revealed that IL32 downregulation increased SPARC protein expression in MPM‐2 cells (Figure S2).

## 4 Discussion

Here, IL32 protein expression was observed in some epithelioid mesothelioma cells, particularly in the papillotubular growth pattern, whereas it was not detected in sarcomatoid mesothelioma cells (Figure 1). Similarly, an IL32 protein band was found in cultured epithelioid mesothelioma cells but not in cultured sarcomatoid mesothelioma cells (Figure 2).

The IL32 gene comprises eight exons and generates nine isoforms through alternative splicing [15]. This study demonstrated that the predominant IL32 isoforms expressed in cultured mesothelioma cells were IL32β and IL32θ (Figure 2). IL32β is mainly localized to the cytosol in endothelial cells [32]. Shim et al. reported that IL32θ had the most dominant biological activity in both immune and non‐immune cells [33]. Similar to IL32β, the IL‐32θ isoform has been proposed to act as an intracellular modulator [33]. Consistently, IL32 protein was not detected in the culture supernatants of mesothelioma cells by immunohistochemical staining (Figure S1).

IL32θ inhibits EMT in HT29 colon cancer cells by suppressing the STAT3‐ZEB1 pathway [34]. In this immunohistochemical study, IL32 was detected mainly in the cytoplasm and partly in the nucleus of mesothelioma tissues (Figure 1a,b). Therefore, it was subsequently investigated whether IL32θ could inhibit the epithelioid‐to‐sarcomatoid phenotypic change in D‐Meso‐Sonobe cells with EMP [24], which refers to the ability of cells to interconvert between epithelial and mesenchymal phenotypes [35]. Notably, IL32θ‐expressing D‐Meso‐Sonobe cells lost epithelial‐mesenchymal plasticity (Figure 3). These findings suggest that IL32θ may inhibit EMT in mesothelioma cells, as observed in colon cancer cells.

Next, the functional association between IL32 expression and mesothelioma cell properties was examined. As shown in Figure 4, IL32 downregulation sensitized both MPM‐2 and TCC‐Meso‐1 epithelioid mesothelioma cells to detachment‐induced apoptosis. These results suggested that IL32 may be linked to anoikis resistance in epithelioid mesothelioma cells.

Anoikis resistance is a key feature of EMT [36, 37]. Notably, enforced IL32θ expression reduced SPARC, a protein recently identified as a driver of EMT in mesothelioma (Figure 3b) [38, 39]. We also found that IL32 downregulation increased SPARC protein expression in MPM‐2 cells (Figure S2). Together, these findings suggest that IL32 may suppress EMT in mesothelioma cells.

Recently, two independent groups reported that IL32 transcripts may serve as a high‐risk biomarker associated with poor prognosis and reduced sensitivity to immunotherapy based on public datasets, including The Cancer Genome Atlas (TCGA) [40, 41]. However, these findings were derived from bulk tissue specimens containing stromal inflammatory cells. Notably, Shi et al. further analyzed these datasets using CIBERSORT, a computational method for estimating cell fractions from bulk tissue gene expression profiles and suggested that the observed IL32 signal may originate from stromal immune cells [41]. We are currently conducting a multicenter study to evaluate the clinicopathological characteristics of IL32‐expressing mesothelioma cells.

Mesotheliomagenesis is closely linked to asbestos‐induced chronic inflammation [42]. Recent advances have highlighted the important roles of IL32 isoforms in the tumor inflammatory microenvironment [15]. For example, IL‐32α promotes a tumor‐supportive microenvironment by inducing inflammation through the NF‐κB signaling pathway in pancreatic cancer [43]. Conversely, autophagy‐related Beclin‐1 limits IL‐32α expression and regulates inflammation in breast cancer stromal niches [44]. IL‐32γ induces COX‐2 expression, which modulates inflammation and contributes to a tumor‐suppressive cancer microenvironment in human papillomavirus‐infected cervical cancer [45]. Although we examined the pathobiological properties of IL32 mainly in relation to EMT, further studies are warranted to clarify the relationship between IL32 isoforms and the inflammatory tumor microenvironment in mesothelioma.

In conclusion, IL32 expression was restricted to a subset of epithelioid‐type mesothelioma cells. IL32θ might attenuate EMT activity in mesothelioma, as previously reported in colon cancer. Moreover, IL32 may contribute to anoikis resistance in epithelioid mesothelioma cells.

## Author Contributions

**Takuya Mikamo:** investigation. **Tamotsu Takeuchi:** conceptualization, funding acquisition, methodology, validation, writing – review and editing, supervision. **Yuki Hanamatsu:** data curation, supervision, resources, visualization, writing – original draft. **Riko Niwa:** investigation.

## Funding

This study was partially supported by the Japan Society for the Promotion of Science KAKENHI (Grant Number 23K06423).

## Ethics Statement

We used commercially available tissue microarrays, which were collected under the Health Insurance Portability and Accountability Act (USA) with informed consent from the donors and in accordance with ethical standards and the Health Insurance Portability and Accountability Act.

## Conflicts of Interest

The authors declare no conflicts of interest.

## Supporting information


**Figure S1:** Cell lysates (lane 1) and culture supernatants (lane 2) from MPM‐2 cells were analyzed by immunoblotting using antibodies against IL32 and intelectin‐1/2. IL32 protein band is observed in cell lysates but not in culture supernatants. Intelectin‐1 is secreted but also found in the cytoplasm of MPM‐2 cells.

**Figure S2:** IL32 protein bands were detected in lysates from negative control siRNA‐treated cells (lane 1) but not in lysates from IL32‐targeting siRNA‐treated MPM‐2 cells (lane 2, #140003; lane 3, #259739). In contrast, SPARC protein bands were present in lanes 2 and 3, but not in lane 1. G3PDH protein bands were detected at similar levels in lanes 1, 2, and 3.

## Data Availability

The data that support the findings of this study are available from the corresponding author, Tamotsu Takeuchi, upon reasonable request.
