# Supplementary figures and images for: Interleukin 32 Expression in Mesothelioma
Source: Thorac Cancer. 2026 Jul 15;17(14):e70353. doi: 10.1111/1759-7714.70353 (PMC13370107; doi:10.1111/1759-7714.70353)

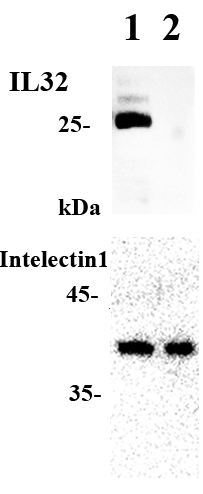

Supplement: Supplementary file 1 — Figure S1: Cell lysates (lane 1) and culture supernatants (lane 2) from MPM‐2 cells were analyzed by immunoblotting using antibodies against IL32 and intelectin‐1/2. IL32 protein band is observed in cell lysates but not in culture supernatants. Intelectin‐1 is secreted but also found in the cytoplasm of MPM‐2 cells. [file TCA-17-e70353-s001.tif]

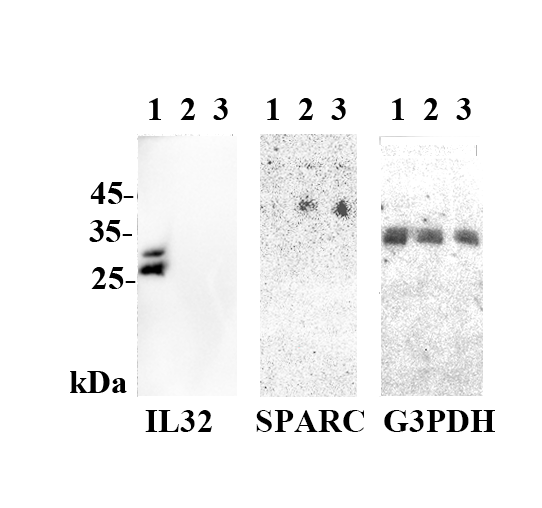

Supplement: Supplementary file 2 — Figure S2: IL32 protein bands were detected in lysates from negative control siRNA‐treated cells (lane 1) but not in lysates from IL32‐targeting siRNA‐treated MPM‐2 cells (lane 2, #140003; lane 3, #259739). In contrast, SPARC protein bands were present in lanes 2 and 3, but not in lane 1. G3PDH protein bands were detected at similar levels in lanes 1, 2, and 3. [file TCA-17-e70353-s002.tif]
